# Twelve tips for designing an inclusive curriculum in medical education using Universal Design for Learning (UDL) principles

**DOI:** 10.15694/mep.2021.000118.1

**Published:** 2021-05-10

**Authors:** Karl Luke

**Affiliations:** 1 Cardiff University

**Keywords:** Universal Design for Learning, inclusive curriculum, curriculum design, inclusivity, medical education, systemic barriers

## Abstract

This article was migrated. The article was marked as recommended.

An inclusive curriculum anticipates and provides strategies to support a diverse range of learners. Universal Design for Learning (UDL) offers medical educators three core principles, which can be used to design curriculum objectives, activities, instructional materials, and assessments with embedded flexibility, equitability, and representation to support diverse learners. Drawing on the available literature and author experience, this article presents twelve tips based on UDL principles for designing and implementing an inclusive curriculum in medical education. This article also questions the purpose of medical curricula and makes recommendations for fostering inclusivity within and beyond the curriculum setting.

## Introduction

Widening access initiatives seek to increase the diversity of learners studying medicine and subsequently diversifying the medical profession ( Patterson and Price, 2017). With such diversification it is essential that measures are introduced to ensure that curriculum design does not produce unnecessary barriers or discriminate against learners with diverse characteristics, backgrounds, circumstances, and preferences. Moreover, designing inclusive and equitable learning experiences is critical to mitigating the predicted global worsening of equity gaps in higher education as a result of the COVID-19 pandemic ( Marinoni, van’t Land and Jensen, 2020; Montacute, 2020).

Curriculum refers to the body of knowledge-content, subjects and/or skills taught in a particular programme, often informed by government, societal, regulatory, and institutional requirements. Curriculum is also concerned with ‘when’ and ‘how’ the overall content will be transmitted or ‘delivered’ to learners ( Smith, 2000). Various interdependent components are involved in the curriculum design process, including the development of learning outcomes, alignment to competence standards, and implementation through teaching, learning and assessment activities ( Morgan and Houghton, 2011). An *inclusive* curriculum anticipates the diverse needs of learners and aims to provide all learners, regardless of their background and immutable characteristics, with equal opportunities to participate fully and achieve the learning outcomes of a programme. Designing an inclusive curriculum should be grounded by the principles of anticipatory, flexibility, collaboration, transparency, and equitability ( McLoughlin, 2001; Morgan and Houghton, 2011).

Principles from Universal Design for Learning (UDL) ( CAST, 2018) provide a useful framework for assisting medical educators in designing programmes with integrated scaffolding of support for learners and flexibility of access to learning with regards to space-time configurations and modality ( Dickinson and Gronseth, 2020). Curriculum designed in conjunction with UDL principles provides flexibility and allows learners to customise their learning experiences to meet their individual needs.

The tips outlined in this article are not presented in any hierarchical order but provide a framework for designing and implementing an inclusive curriculum in medical education. The guidance also explores the important issue of social inequality in relation to curriculum design and makes recommendations for fostering inclusivity using UDL principles.

## Tip 1: Embed inclusive design from the start

Many countries have equality legislation in place which requires educators to make reasonable accommodations or adjustments to ensure that learners are not discriminated in relation to disability and protected characteristics. However, inclusive curriculum design should anticipate and reduce the need for individual adjustments ( Bunbury, 2020). Where individual accommodations remain necessary, it is recommended they are designed and implemented in partnership with the learner. Moreover, engaging learners throughout the curriculum design process can help educators anticipate potential adjustments and help evaluate whether such accommodations could benefit a larger population of learners and become mainstreamed ( Morgan and Houghton, 2011).

Universal Design for Learning (UDL) ( CAST, 2018) is a set of principles, grounded by cognitive neuroscience, for designing curriculum that aims to provide all individuals with equal learning opportunities, regardless of (dis)ability, gender, age, or cultural background. The three principles of UDL are:

1. **Multiple means of representation:** Using a variety of strategies to present information; providing a range of methods to support perception and comprehension.
2. **Multiple means of action and expression:** Providing diverse learners with alternative ways to act competently; providing alternatives for demonstrating what learners have learned.
3. **Multiple means of engagement:** Aligning to learners’ interests by offering choices of modality, content, and tools; optimising relevance, value, and authenticity; motivating learners by offering variable levels of challenge and effective feedback.

UDL is significantly different from other curriculum design approaches as it encourages educators to undertake the design process by expecting the curriculum to be accessed by diverse learners with varying abilities and skills. This anticipatory design approach encourages educators to design curriculum that recognises and embraces diversity in learners’ needs and preferences ( Sanger, 2020).

## Tip 2: Define clear and achievable learning outcomes

In establishing curriculum objectives it is important that they are aligned to professional competence standards and are also designed to allow learners multiple ways to demonstrate goal achievement. This advocated by the UDL principle of multiple means of *action and expression.*

Effective inclusive curriculum design, including the teaching strategies, materials, and assessment design, involves flexibility and strong constructive alignment with the intended learning outcomes ( McLoughlin, 2001). Learning outcomes should be non-discriminatory by design and reflect a genuine measure of achievable competence ( Morgan and Houghton, 2011). As an example, it is important to design goals and strategies within the medical curriculum that will enable all learners to have equitable and flexible opportunities to develop and express pre-clinical knowledge, competencies, and attributes. Simulated, virtual, or augmented activities potentially provide a safe environment for learners to practise, acquire, and enhance skills required during clinical practice ( McLean and Gibbs, 2010; Dickinson and Gronseth, 2020). Whilst such methods are likely to benefit a range of learners, they may be particularly helpful for the unconfident learner or those with restricted opportunities to acquire skills before practical training starts ( Morgan and Houghton, 2011). This is a particularly important consideration given the restrictions forced upon some training opportunities by the COVID-19 pandemic. Therefore, when designing learning outcomes carefully consider how a diverse cohort of learners can achieve and express these.

Learning outcomes should be clearly presented to learners within the syllabus. A syllabus is typically presented as a document which outlines the topic or concept areas that may be assessed and examined and is often developed by the educator. It provides learners with the required elements of a course or programme and can shape learner expectations ( Harnish and Bridges, 2011). For educators, it serves as an instrument to help plan and organise the content and activities that learners must engage with during a course ( Slattery and Carlson, 2005). Language used with the syllabus can influence learners’ initial impressions of educators. Incorporating friendly language and a pleasant tone within the syllabus can foster learner motivation and positive relationships between faculty and learners ( Harnish and Bridges, 2011).

## Tip 3: Diversify the curriculum

A goal of inclusive curriculum design is to recognise, value, and integrate the diverse identities within a programme, which is supported by the UDL principles of multiple means of *representation* and *engagement.* Particularly important when teaching diverse learners from different backgrounds, countries, or cultural traditions, proactively diversifying the curriculum will help learners feel that they belong, which can be a powerful motivator for engagement and learning ( Sanger, 2020).

Review the curriculum so it does not perpetuate stereotypes and explore if bias is evident within the content and activities. Does the curriculum preference certain groups, contexts, mindsets, or cultures? For example, the UK medical curriculum has been criticised for lacking cultural diversity and offering traditionally white, androcentric, Eurocentric content ( Gishen and Lokugamage, 2019). Curriculum designers should actively explore unconscious biases within the curriculum and prevailing forms of privilege ( McIntosh, 1989).

Importantly, there is growing concern that the medical curricula offered by UK Universities marginalises and alienates minority learners. In recent years, calls have intensified for UK universities to “decolonise” the curriculum and rebalance the dominance of western values and beliefs ( Nazar *et al.,* 2015; Lokugamage, Ahillan and Pathberiya, 2020). Educators should endeavour to remove colonial references and design a curriculum which allows learners to see themselves and others exhibited in positive ways. There are recent examples of medical schools in the UK actively widening the curriculum to include teaching on racial bias within medical trials and the historical exploitation of black people in medical research, and training on topics such as spotting unconscious bias and racism ( Mundasad, 2020). Verdonk and Janczukowicz (2018) observe that as patient populations become increasingly varied and complex, educators need to equip medical students with the attributes, knowledge, and skills to treat diverse patients fairly and non-judgmentally. Gishen and Lokugamage (2019) also argue that by introducing diversity related topics into the medical curriculum increases medical students’ confidence, communication skills, and potentially improves future patient care.

## Tip 4: Co-design with learners

The UDL principle of providing multiple means of *engagement* centres around the concept of varying learner motivations and educators should provide a variety of ways to focus and engage learners. To help foster learner engagement, medical educators can actively involve learners in aspects of curriculum design by offering opportunities to inform the development of learning outcomes, the types of resources used, the subjects studied, or the modalities of assessment.

Learners can act as powerful change agents through co-producing medial curricula ( Burk-Rafel *et al.,* 2020). Engaging learners as authentic partners in the curriculum design process allows greater ownership, accountability, and understanding about the purpose and context of the learning experience ( Morgan and Houghton, 2011). Engaging learners as curricula co-creators may produce more learner-centred and equitable educational programs, whereby more diverse voices are represented ( Burk-Rafel *et al.,* 2020). There is evidence of medical educators actively engaging learners as stakeholders in curricula reforms, whereby learners are provided opportunities to critically question and challenge inequalities and gaps inherent in their curriculum and develop innovative approaches to address such challenges ( Krishnan *et al.,* 2019; Moss *et al.,* 2020). Within the curriculum, educators could also explore principles of *allyship* ( Ng, Ware and Greenberg, 2017) and encourage learners to co-design practical strategies for enacting equitable relationships with peers inside and beyond the curriculum setting ( Ackerman-Barger *et al.,* 2020; Roberts, 2020).

## Tip 5: Present diverse voices and perspectives

Educators can promote a sense of belonging for learners by considering learner diversity and offering underrepresented scholarly perspectives. This could be achieved by ensuring diversity of perspectives within reading lists, by promoting authors of different gender identities, or incorporating case studies from a variety of regions and countries ( Sanger, 2020). Doing so supports the UDL principle of providing multiple means of *engagement* by aligning to learners’ interests and motivations, as well as providing authentic experiences. If offering broad and diverse representation is challenging within a discipline, acknowledge imbalances and encourage critique. If a seminal text only uses male pronouns, or stereotypical case studies, acknowledge this and give learners an opportunity to discuss and critique. This can help learners see how and why particular materials or case scenarios are selected. For example, if all essential texts are androcentric and produced in Europe or North America, discuss this with the learners and explain the rationale behind their inclusion. Some key medical concepts may be strongly associated with white males, which could be addressed by arranging seminar speakers from diverse backgrounds to present content and to provide learners with positive role models from marginalised communities.

Where possible, underrepresented communities, such as the LGBT+ community and black, Asian and minority ethnic (BAME) groups, should be visible within the curriculum, which will enable learners and educators to feel less marginalised ( Gishen and Lokugamage, 2019). Ensure a range of examples from diverse communities are provided in lecture content, reading lists, and problem-based scenarios. For example, LGBT+ case inclusion and providing illustrative photos of clinical signs on both light and dark skin tones. It is important to present diverse communities positively, equally, and avoid stereotyping; this is particularly important as stereotyping can have a profound negative impact on learning and performance ( Steele and Aronson, 1995). To promote an environment of belonging and respectful inclusion, develop a mutually agreed charter - or ground rules - between learners and educators outlining expected behaviours and responsibilities within all learning spaces (including virtual spaces). To further support a sense of belonging, in discussions - whether online or in-person - apply the preferred names and pronouns used by learners.

## Tip 6: Review the timetable and delivery

When considering the UDL principle of *action and expression,* educators should provide learners with multiple means to interact with the curriculum, including the resources, materials, tools, technologies, and peers. Review the curriculum timetable and critically consider if the timing of the teaching and learning activities might negatively impact some learners (e.g. religious or cultural holidays and celebrations). Using an online diversity and inclusion calendar can be helpful in planning activities.

Where possible, curricula should provide opportunities for flexible delivery using both asynchronous and synchronous online activities, which removes some of the barriers associated with developing a fixed timetable and conflicts with clinical responsibilities and rotas ( McLean and Gibbs, 2010; Dickinson and Gronseth, 2020). *Synchronous* learning refers to learner engagement with materials, instructors, and peers in real-time, although not necessarily in the same place, such as through the use of virtual classroom technologies. This contrasts to *asynchronous* learning, which does not involve learners in the same place or at the same time (e.g. a task to gather information on a topic individually by a set date).

## Tip 7: Design opportunities for cooperative learning

Creating peer learning opportunities helps exploit the advantages offered by a diverse cohort of learners and supports the UDL principles of providing multiple means of *engagement* and *expression/action.* Learners often value opportunities to share their background and perspectives, therefore design activities and assessments that encourage peer working and support. Design opportunities within the curriculum which allow learners to bring together their unique voices for shared enterprise, for example through cooperative learning which prioritises collaboration to achieve learning goals. Techniques, such as the Jigsaw method, can be a successful strategy in which small groups, with mixed levels of ability, use a variety of activities to develop joint understandings of a topic area or subject ( Walker, Olvet and Chandran, 2015; Eachempati, KS and Ismail, 2017). Creating space within the curriculum for learners to share personal experiences can support learner motivation and sets an inclusive tone by encouraging each learner to share understandings. When designing small group work activities, ensure that the allocation of learners enables the formation of ethnically diverse groups from various educational backgrounds.

## Tip 8: Consider effective teaching strategies

There is some evidence to suggest that learner-centred strategies may have a negative impact on learners from lower socio-economic backgrounds and may perpetuate some inequalities in education ( Andersen and Andersen, 2017). Furthermore, as the majority of medical students may have relatively similar pre-entry qualifications, there can be an implicit assumption that these learners will share similar learning experiences, approaches, and preferences ( Morgan and Houghton, 2011). However, this is unlikely to be the case and learners deploy a range of strategies depending on their abilities, needs, and preferences. The UDL principle of *representation* advocates that educators should guide information processing and engage learners by activating prior knowledge. Incorporating different teaching strategies and varying activities will offer a more inclusive experience for learners with different prior knowledge, experiences, and learning needs. Therefore, when designing opportunities for self-directed learning or collaborative learning, medical educators have an important role in providing *direct instruction,* which involves providing clear and detailed instructions, guiding learners as they begin independent practice, and offering examples and explanations ( Kirschner and Hendrick, 2020).

UDL principles advocate that medical educators should avoid or explain culturally specific references, clarify medical vocabulary and symbols, and provide cognitive supports in instruction. Giving learners organising clues can be an effective strategy, for example: “ *we have explored four risk factors associated with disease progression, which I will now summarise.*” Provide background or framing information for new concepts using artefacts such as images, articles, and videos, which can be explored independently outside of a teaching session. Scaffold learning (offer guidance to reduce the complexity of a task) by providing access to resources (e.g. syllabus, summaries, study guides, PowerPoint slides) and tutor support (e.g. formative assessment feedback, tutorials) ( Sanger, 2020). Clearly segment teaching sessions and activities into chunks, which will help learners to organise content into coherent cognitive structures ( Mayer, 2010). Design learner activities that require retrieval practice and provide worked examples, demonstrating how to solve a specific problem or modelling expected outcomes, which can be effective for optimising germane load ( Collins *et al.,* 2020).

## Tip 9: Offer multiple strategies to present information

The UDLprinciple of *representation* advocates that educators should provide instruction through multiple forms of media and provide a variety of ways to interact with educational content to generate new understandings. Enhance instruction through a range of activities, such as the use of case studies, poetry, patient stories, stimulations, role play, practical activities, guest speakers, virtual communications, and educational software. Within the curriculum offer alternative learning contexts by designing opportunities for individual and collaborative working, as well as distance learning, peer learning, and clinical work.

Teaching and learning materials should be presented in a range of modalities, such as online resources, digital learning objects, videos, articles, podcasts, PowerPoint presentations, and e-books. When designing multimedia artefacts and presentations critically consider effective design principles ( Pate and Posey, 2016). Mayer’s (2010) multimedia design principles offer practical guidance for reducing extraneous information, such as decorative images, and optimising the presentation of multimodal aspects for efficient cognitive processing and learning.

Where possible, provide instructional material in alternative formats. For example, provide access to both physical and electronic versions of key textbooks, capture lectures using recording software, and provide a digital document version of online learning packages (e.g. a downloadable PDF file). Providing captions to videos and podcasts, or enabling live captions within online synchronous tools (e.g. Microsoft Teams), serves the dual purpose of enhancing accessibility for learners with hearing impairments and supporting multiple options for perception, which aligns with the UDL principle of *representation* and can be particularly beneficial for second language learners ( Dickinson and Gronseth, 2020). This offers an example of how adjustments can become mainstreamed to benefit a wider group of learners.

## Tip 10: Use technology appropriately

Whilst not an inherent solution, if used appropriately technology can be used to facilitate enhancements within inclusive curriculum design and is highlighted throughout the three core principles of UDL. For example, the use of touch-sense haptic technologies and stimulations can replicate learning experiences that might otherwise be inappropriate for learners to undertake on real patients, for example based on training stage or issues with access to assessors for appraisal ( Morgan and Houghton, 2011). Such technologies also allow greater flexibility with regards to the space-time dimensions of learning. UDL principles have also been successfully deployed in clinical practice with the support of technologies ( Martyn, Pace and Gee, 2015). In practice environments, mobile technologies and assistive applications have been used to provide feedback (e.g. mini-CEX) and electronic note-taking whilst on ward rounds ( Morgan and Houghton, 2011). Developing clear policies and processes (e.g. data protection and confidentiality) that break down the barriers to using technology, including the use of mobile devices and assistive applications in clinical environments, helps foster an enabling learning experience ( Martyn, Pace and Gee, 2015).

To ensure the inclusive use of technology, potential barriers - such as skills, ability, equipment access, financial, and accessibility - need consideration prior to implementation. Importantly, the use of technology should not be a barrier for learners with disabilities ( Hersh and Mouroutsou, 2019). For example, medical educators can make simple adjustments when designing resources and multimedia content by using consistent page titles and headings, and images should be relevant to the content and include alt text (alternative text descriptions). This is crucial for learners who rely on assistive technologies, such as screen readers, to interact with content. Consistent and clear page titles provides clarity and structure to assist learners with orientation and navigation, and alt text within images are used by applications to describe images to learners with visual impairments.

## Tip 11: Provide flexible opportunities for assessment and feedback

UDL principles advocate that the curriculum should provide multiple means of learner *action and expression,* whereby learners are offered alternative means to demonstrate what they have learned ( Sanger, 2020). Medical educators should design different assessment options to demonstrate learning, such as oral presentations (individual or team-based), written assignments (e.g. essays, fictional, creative), or visual representations (e.g. posters, videos). Where summative assessments, such as traditional essays or exams, are obligatory due to professional body requirements, different formative assessment options should be offered, such as more personalised submissions (e.g. reflective commentaries, online discussion forums, blogs) ( Morgan and Houghton, 2011).

Feedback is an essential component of learning and its value is widely recognised within medical education ( Bing-You *et al.,* 2017; Kornegay *et al.,* 2017). Within the curriculum, design opportunities for formative feedback based on group and individual activity. When considering effective feedback in diverse contexts, ensure feedback is timely, honest, direct, and constructive. Recommend concrete steps for improvement and signpost additional resources and services which can be accessed flexibly. Support learner motivation by providing feedback, setting goals, and providing rewards for completing tasks. Importantly, the UDL *engagement* principle also recommends that educators support learners in developing self-regulatory skills and provide opportunities for learner self-assessment and self-reflection.

## Tip 12: Evaluate curricula using an inclusive design checklist

As discussed, an inclusive curriculum anticipates and provides strategies to support a diverse range of learners. While UDL itself is not a list of strategies that must be implemented, the UDL Guidelines ( CAST, 2018) offer a set of practical suggestions that can be applied to any discipline and provides ideas for UDL implementation. To help with the implementation of UDL principles, many UDL checklists can be found online, such as those designed by Iowa State University (2019) and West Virginia Department of Education (Unknown). Such UDL checklists can be a useful starting point in evaluating the current level of inclusively with programmes or for planning curriculum changes ( Bartholomew and Griffin, 2018).

Many UK Universities have also designed general inclusive curriculum checklists. Whilst not explicitly aligned to the UDL principles, many of the checklists offer important aspects covered by UDL. Examples of inclusive curriculum checklists can be found from University of Dundee (2017), University College London (2018), and Manchester Metropolitan University (2020). Importantly, inclusive curriculum checklists can be completed as part of the development and design of new programmes and the periodic review of existing programmes.

## Conclusion

Medical curricula are not static and continues to evolve to reflect new ideas, practices, and knowledge. Designing an inclusive curriculum extends to critically exploring the ideological assumptions underpinning the purpose of the curricula ( Smith, 2000). Within medical education, outcomes‐based curricula, which is heavily dependent on the setting of behavioural objectives, has been challenged by educators who argue that learning outcomes are unable to accurately specify educational achievements and the true effects of learning ( Rees, 2004). Scholars have advocated a paradigm shift from *curriculum as product* to emancipatory models, known as *curriculum as praxis,* which focuses on the domains of knowledge, critical reflection, and committed action ( Ford and Profetto-McGrath, 1994). The curriculum as praxis model is concerned with the development of a critical consciousness, and actively seeks to challenge inequalities through action. For example, Shahvisi (2019) reports on teaching UK medical students of the significant social status and privilege they will have as doctors, and the importance of collective action against damaging agendas and social injustices. Moreover, it is essential that medical curricula attend to the current political, economic, and societal issues we face, including global pandemics, social injustices, technological advances, and climate change ( Verdonk and Janczukowicz, 2018; Finkel, 2019; Goh and Sandars, 2020).

Implementing curriculum changes poses numerous challenges such as stakeholder resistance, lack of skills and knowledge, time pressures, lack of desire or damaging conflicts, and issues with communication and planning ( Luke, 2021). Adopting a methodological approach to managing curriculum change is essential and awareness of the need for change is a key foundation in the change process ( Luke, 2021). This paper has aimed to raise awareness of the importance of inclusivity within medical education and explored how UDL principles can be used to develop a flexible curriculum, which embeds opportunities for learners to be active participants in the curriculum design and delivery. The three core UDL principles offer medical educators opportunities to design curriculum outcomes, activities, instructional materials, and assessments with embedded flexibility, equitability, and representation to support diverse learners in any context. In turn, an inclusive curriculum may help in tackling systemic barriers that produce inequitable learning opportunities and outcomes. Inclusive curriculum design may also provide learners with the critical skills and competencies to positively contribute to diverse workplaces and wider society.

## Take Home Messages

• Use UDL principles when (re)designing curriculum. Flexibility, variation, and engagement are key principles for universal design.
• Recognise, incorporate, and celebrate diversity in the curriculum. Demonstrate the cultural and social relevance of course concepts.
• Design opportunities for personalising activities and allow learners to co-design elements of the curriculum.
• Use technology appropriately and digitise resources. Present content in alternative formats (text, audio, and/or visual). For example, record lectures where possible.
• Use varied teaching methods and provide learners with active learning opportunities. Use alternative strategies for assessing learning.

## Notes On Contributors

**Karl Luke** is a lecturer in Medical Education at Cardiff University, United Kingdom. Karl is a Senior Fellow of the Higher Education Academy (SFHEA) and Certified Member of the Association of Learning Technology (CMALT). Karl’s research interests include digital education, multimodality and sociomateriality. ORCiD: https://orcid.org/0000-0002-7765-6126
