# Supplementary material for: Twelve tips for designing an inclusive curriculum in medical education using Universal Design for Learning (UDL) principles
Source: MedEdPublish (2016). 2021 May 10;10:118. [Version 1] doi: 10.15694/mep.2021.000118.1 (PMC10939519; doi:10.15694/mep.2021.000118.1)
Supplement: Peer review history

# Reviewer report: Reviewer response for version 1

**DOI:** 10.21956/mep.18913.r27032

**Published:** 2021-10-09

**Authors:** Anita Samuel

This review has been migrated. The reviewer awarded 4 stars out of 5 This is a practical and informative article that is relevant as we address issues of equity in medical education. It is nice to see UDL being introduced to medical education curriculum. You have provided some practical strategies that can be immediately implemented. Below are some suggestions to strengthen this article:• Framing UDL as a part of curriculum (re)design can be a daunting undertaking. It might be helpful to mention that UDL principles can be incorporated into existing curricula. This makes it much more accessible for meded programs. • The last paragraph of Tip 2 focuses on syllabus. It is important information but is not really connected to the theme of the tip. • The first sentence in Tip 6 also appears out of place.

Reviewer Expertise:

NA

No decision status is available

# Reviewer report: Reviewer response for version 1

**DOI:** 10.21956/mep.18913.r27031

**Published:** 2021-10-06

**Authors:** Richard Hays

**Affiliations:** 1 James Cook University

This review has been migrated. The reviewer awarded 3 stars out of 5 I found this a difficult article to review. The author has used a curriculum development framework developed in California and has applied this to undergraduate medical curriculum design in the UK context, offering 12 tips for that may improve curriculum inclusivity and diversity. I will come back to the tips but first, I have two concerns that I would like to discuss with the author, but will have to do this the slow way via this review. The first is that I am not sure that the case for such a potentially major change in overall curriculum design and delivery is made. Whenever I have been involved in curriculum design, I have stuck by the rule that for something else to go in, something must come out. Medical curricula are already so overcrowded in both content and delivery that I would not support adding to it. It is difficult to argue for a further reduction in scientific content and there already are curriculum domains that include communication skills, and personal and professional behaviours. There may be scope to develop further those domains and I know that many medical programs are moving in that direction. The second concern is that I am not sure that the UDL framework is valid for tertiary professional education. It seems more relevant to primary and secondary education for populations where there are substantial minority groups that face substantial disadvantage. Widening participation is an important and necessary strategy, but the frameworks may be best applied in precursor programs and pathways so that medical school applicants are better prepared and more likely to be successful in medical programs. Following the UDL principles may be even more important early in the education system to bring and retain more people from minority populations in the high achiever group that might consider medical careers. Coming back to the 2 tips, I think that these are sensible, because the dual aim of curriculum design is to ensure that graduates meet community expectations. These expectations are high wrt knowledge, skills and professional behaviours, but can underlying attitudes be changed by simple inclusion strategies? Adult behaviours are a product of their family background and life experience (with some genetics in there somewhere) and so the content of a medical curriculum may be only a small influence on the end product. More important is role modelling, and there is already evidence that the health system has many poor role models who may normalise poor behaviours. Overall I found this a really thought-provoking article, although feel that more work needs to be done on why and how to to apply important contemporary issues in a practical way. Despite my reservations I would recommend this be read by anyone involved in curriculum management. It would be good to have the opportunity to discuss these issues - the topic may be appropriate for a conference symposium.

Reviewer Expertise:

NA

No decision status is available
